# One-year effects of two intensive inpatient treatments for severely obese children and adolescents

**DOI:** 10.1186/s12887-016-0659-x

**Published:** 2016-08-02

**Authors:** Sabine Makkes, Carry M. Renders, Judith E. Bosmans, Olga H. van der Baan-Slootweg, Trynke Hoekstra, Jacob C. Seidell

**Affiliations:** 1 Department of Health Sciences and the EMGO Institute for Health and Care Research, VU University Amsterdam, Amsterdam, The Netherlands; 2 Merem Childhood Obesity Center, Heideheuvel, Hilversum, The Netherlands; 3 Department of Epidemiology and Biostatistics, VU University Medical Center, Amsterdam, The Netherlands; 4 Department of Health Sciences, Faculty of Earth and Life Sciences, Section Prevention and Public Health, VU University Amsterdam, De Boelelaan 1085, 1081 HV Amsterdam, The Netherlands

**Keywords:** Morbid obesity, Child, Adolescent, Lifestyle, Treatment effectiveness

## Abstract

**Background:**

Intensive inpatient lifestyle treatment may be a suitable alternative for severely obese children and adolescents who do not benefit from ambulatory obesity treatment.

The aim was to evaluate the effectiveness of two intensive one-year lifestyle treatments with varying inpatient periods for severely obese children and adolescents with regard to SDS-BMI and cardiometabolic risk factors.

**Methods:**

The study was designed as a randomized controlled trial with two active treatment groups.

Eighty participants (8–19 years) with severe obesity received treatment at a specialized childhood obesity center in the Netherlands. Severe obesity was defined as a SDS-BMI ≥ 3.0 or a SDS-BMI ≥ 2.3 in combination with obesity-related comorbidity.

Participants received an intensive one-year lifestyle treatment with an inpatient period of either two months and biweekly return visits during the next four months (short-stay group) or six months (long-stay group), both followed by six monthly return visits.

Outcomes were assessed at baseline, six and 12 months and included SDS-BMI as primary outcome and cardiometabolic risk factors such as SDS-waist circumference, systolic- and diastolic blood pressure, and blood measurements as secondary outcomes.

To evaluate differences in the course of the primary- and secondary outcomes over time between the two treatment groups, Generalized Estimating Equations (GEE) were performed.

**Results:**

No differences in the course of SDS-BMI or secondary outcomes over time were found between the two treatment groups after one year of treatment. SDS-BMI decreased statistically significantly after one year of treatment compared with baseline in both groups (0.33 (0.48) in the short-stay and 0.52 (0.49) in the long-stay group). Similar results were found for SDS-waist circumference, diastolic blood pressure and HDL-cholesterol.

**Conclusions:**

Since there were no significant differences in effects between the short- and long-stay treatment and considering the burden of the long-stay treatment for children and families, we recommend implementation of the short-stay treatment.

**Trial registration:**

Netherlands Trial Register NTR1678, registered 20-Feb-2009

## Background

During the last few decades, the prevalence of (severe) obesity in children and adolescents has been rising worldwide [1–5]. In the Netherlands, the upward trend of severe childhood obesity resulted in a seven-fold increase in the prevalence between 1980 and 2010. It was estimated that in 2010 the prevalence was 0.56 % (which corresponds to approximately 18 500 children and adolescents in the country) [6].

Childhood obesity increases the risk of cardiometabolic risk factors such as hyperlipidemia, hypertension and diabetes mellitus type 2, as well as respiratory and musculoskeletal conditions and liver abnormalities [7, 8]. Previous studies have shown that 62-80 % of severely obese children and adolescents have at least one cardiometabolic risk factor in addition to being severely obese [9, 10]. Moreover, (severe) obesity has a negative impact on psychosocial health [11–14]. Severely obese children reported to have a similar quality of life as children diagnosed with cancer [15]. Furthermore, there is a high probability that childhood obesity tracks into adulthood leading to health problems later in life [16, 17].

Research shows that obesity treatment should incorporate a combination of changes in diet and physical activity, and needs to be family-based [18–22]. Several studies have shown that obesity treatment can lead to weight loss in obese children and adolescents, and that this may reduce the serious immediate and long-term burden on physical and psychosocial health for these obese individuals and society as a whole [23–25]. Research in severely obese children and adolescents is relatively rare; most studies included children with lesser degrees of obesity. Evidence suggests that ambulatory obesity treatment for severely obese children and adolescents is insufficiently effective in the long-term and that more intensive treatment in specialized centers is needed [22, 26–28].

Currently the treatment center “Heideheuvel” is the only specialized childhood obesity center in the Netherlands that offers treatment for severely obese children and adolescents. Their intensive one-year lifestyle treatment was modeled after the treatment programme developed by Braet et al. [29] and originally included a six-month inpatient period. This treatment proved to be more effective in improving SDS-BMI and cardiometabolic risk factors than ambulatory obesity treatment [30]. However, an inpatient period of six months is expensive and poses a considerable burden on both the participants and their families. Therefore, a modified treatment was developed with a two-month inpatient period [31]. The aim of this study was to evaluate the effectiveness of two intensive one-year lifestyle treatments with varying inpatient periods (i.e. two months vs. six months) for severely obese children and adolescents with regard to SDS-BMI and cardiometabolic risk factors such as SDS-waist circumference, systolic- and diastolic blood pressure, and blood measurements directly after treatment.

## Methods

### Study design and population

This study was designed as a randomized controlled trial with two active treatment groups receiving a one-year treatment programme which included a two months inpatient part in one group and six months inpatient part in the other group. There was also a waiting list controlgroup. This paper reports on the effectiveness of the two treatments directly after one year of treatment. The Medical Ethics Committee of the VU University Medical Center (Amsterdam, the Netherlands) approved the study protocol. Prior to randomization, written informed consent was obtained from both the participants and their parents/caregivers. Details of the study have been described elsewhere [31].

The study population consisted of 80 participants (8–19 years) with severe obesity. All participants were referred to a specialized childhood obesity center by their local pediatrician after insufficient response to ambulatory obesity treatment. Severe obesity was defined as a SDS-BMI ≥ 3.0 (99.9th age- and sex-specific percentile of BMI in the fourth Dutch nationwide growth study of 1997), or a SDS-BMI ≥ 2.3 (99th age- and sex-specific percentile of BMI in the fourth Dutch nationwide growth study of 1997) in combination with obesity-related comorbidity. Participants were excluded from the study if they had syndromal or chromosomal determined obesity; obesity caused by endocrine abnormalities or medicine use; psychiatric problems; an IQ below 75 or if their parents/caregivers were not willing or able to participate in the treatment and/or study [31].

### Intervention conditions

Both groups received an intensive one-year lifestyle treatment with either an inpatient period of two months (short-stay group) or six months (long-stay group). During weekdays, the short-stay group participated in a two-month inpatient treatment, followed by biweekly return visits of two days during the next four months, then followed by six monthly return visits of two days. The long-stay group participated in a six-month inpatient treatment during weekdays, followed by six monthly return visits of two days. The treatment focused on nutrition, physical activity and behavior change and required active participation of the parents/caregivers. Treatment was delivered at a specialized childhood obesity center, Heideheuvel, in the Netherlands. A more detailed description of the content, frequency and intensity of the treatment can be found elsewhere [31].

### Randomization and blinding

The primary researcher, who was not blinded to treatment allocation, randomized all participants to the short-stay (40 participants) and long-stay group (40 participants) using a table of random numbers [32, 33]. Because it was logistically not possible to provide treatment to all participants at the same time, a group of 20 participants was randomized to a one-year waiting-list group, after which they were randomly allocated to one of the treatment groups. Four participants dropped out of the study while being in the waiting-list group, leading to a waiting-list group of 16 participants. Four additional participants were recruited to replace the four participants who dropped out of the study to ensure a study population of 80 participants.

Because of the nature of the treatment, participants, their parents/caregivers and healthcare professionals could not be blinded to the treatment.

### Measurements

Demographic characteristics included ethnicity, educational level, socio economic status (SES) and household situation [31].

Outcome measures were collected at baseline and six and 12 months of follow-up. SDS-BMI was the primary outcome in this study and cardiometabolic risk factors (i.e. SDS-waist circumference, systolic- and diastolic blood pressure, and blood measurements) the secondary outcomes.

BMI was calculated as weight/height^2^ (kg/m^2^). The degree of overweight was quantified using Cole's least mean square method, which normalizes the BMI's skewed distribution and expresses BMI as SDS-BMI [34]. SDS-BMI was calculated with the Growth Analyser [35] using the fourth Dutch nationwide growth study of 1997 as reference.

Waist circumference was measured with a tape measure with an accuracy of 1 mm. SDS- waist circumference was calculated with the Growth Analyser [35] using the fourth Dutch nationwide growth study of 1997 as reference.

Blood pressure was measured three times in sitting position after sitting still for at least 5 min. For the analyses, the averages of the three systolic blood pressure and diastolic blood pressure readings were used.

Blood measurements included fasting insulin, 2h-insulin, fasting glucose, 2h-glucose, HDL-cholesterol, triglycerides and homeostasis model assessment for insulin resistance (HOMA-IR) after an overnight fast [36].

### Statistical analyses

The sample size was calculated to detect a 0.5 SDS-BMI difference between the two groups after one year of treatment which is considered a clinically meaningful effect size [37]. Based on a Power of 80 % and a two-tailed significance level of 5 %, two groups of 40 participants were needed [31].

Analyses were performed according to the intention-to-treat principle. Baseline characteristics were compared between the two treatment groups. Independent Student’s t-tests were used for continuous variables and Chi-square tests for categorical variables using IBM SPSS Statistics for Windows, Version 21 (SPSS 21) [38]. Statistical significance was set at a two-sided *P*-value < 0.05.

To evaluate differences in the course of the primary- and secondary outcomes over time between the two treatment groups, Generalized Estimating Equations (GEE) were performed [39]. GEE were used to adjust for the correlation between repeated measures obtained in the same participant. In all models, an exchangeable correlation structure was specified and adjustment for baseline values was applied to assess the actual effects of treatment on the primary- and secondary outcomes independent of differences in baseline values [39]. To evaluate the effects of the two treatment groups at different time points specifically (i.e. between baseline and six months follow-up and between six- and 12 months follow-up, respectively), time was treated as a categorical variable according to the common approach described earlier by Fitzmaurice et al. [40].

For each outcome, two types of analyses were performed: 1) crude analyses which were only adjusted for baseline values and 2) analyses which were adjusted for baseline values and additional covariates.

Covariates were selected by first assessing them using a forward approach and were considered relevant when the treatment effect changed with 10 % or more after inclusion of the covariate [41]. All covariates were also tested for possible effect modification and if the interaction term was statistically significant (i.e. a *P*-value ≤ 0.05), stratified models were presented.

### Additional analyses

To evaluate the effectiveness of the two treatments in comparison with the waiting-list group in the course of the primary- and secondary outcomes over time, GEE were performed as well [39].

In the per protocol analysis, only participants who took part in at least 75 % of the treatment sessions were included. In the complete case analysis, only participants with complete follow-up on the primary outcome SDS-BMI (baseline, six and 12 months) were included. In both the per protocol and complete case analyses, the primary- and secondary outcomes over time were evaluated with GEE.

## Results

### Participants

**Fig. 1:**
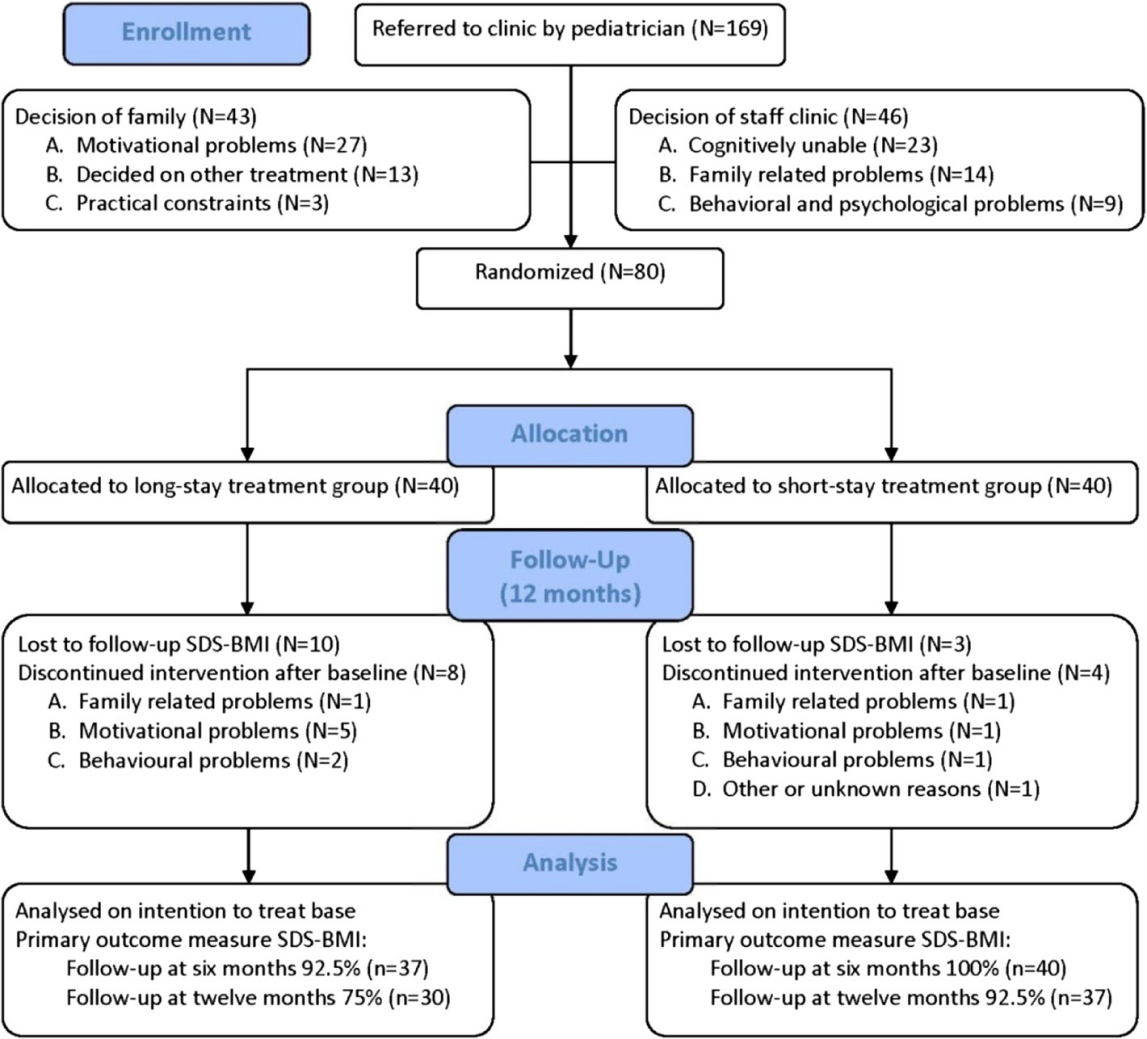
Flow-diagram of participants. A one-year waiting-list group of 20 participants was involved. Four participants dropped out of the study while being in the waiting-list group, leading to a waiting-list group of 16 participants

In total, 169 participants were referred to Heideheuvel by their local pediatrician after insufficient response to ambulatory obesity treatment. Of them, 89 were excluded based on either a decision made by the staff of Heideheuvel (*N* = 46) or by the family (*N* = 43). This left 80 participants to be included in the study (Fig. 1).

At baseline, no relevant differences were found between the two treatment groups (Table 1). Sixty-eight participants (85 %) completed the treatment programme. Of these 68 participants, 61 participants (76 %) took part in at least 75 % of the treatment sessions and were considered as per protocol participants. Complete follow-up on the primary outcome SDS-BMI was obtained from 37 short-stay and 30 long-stay group participants (84 %). Per protocol and not per protocol participants, and participants with and without complete follow-up did not differ from each other with regard to baseline characteristics.

**Table 1:** Baseline characteristics of the study population

	Total	Short-stay group	Long-stay group
	*N* = 80	*N* = 40	*N* = 40
Age (years) [mean (SD)]	14.8 (2.3)	14.5 (2.4)	15.0 (2.2)
Female [n (%)]	53 (66.3)	28 (70.0)	25 (62.5)
Ethnicities [% of total]			
Western	61.5	69.2	53.8
Non-Western	38.5	30.8	46.2
Educational level of the parents/caregivers [% of total]^a^			
Low	38.7	38.5	38.9
Medium/intermediate	42.7	43.6	41.7
High	18.7	17.9	19.4
SES [% of total]			
Below average	65.8	59.5	71.8
Above average	34.2	40.5	28.2
Household situation [% of total]			
Married/living together	55.0	62.5	47.5
Divorced	33.8	32.5	35.0
One parent family(mother)	7.5	2.5	12.5
Other situation	3.8	2.5	5.0
SDS-BMI [mean (SD)]	3.4 (0.39)	3.4 (0.39)	3.4 (0.39)

The short-stay group participated in a two-month intensive inpatient treatment during weekdays, followed by biweekly return visits of 2 days during the next 4 months, then followed by six monthly return visits of 2 days. The long-stay group participated in a six-month intensive inpatient treatment during weekdays, followed by six monthly return visits of 2 days

*Abbreviations*: *SD* standard deviation, *SDS-BMI* standard deviation of body mass index, *SES* socio economic status

^a^Educational level was classified according to the definition of Statistics Netherlands (http://www.cbs.nl)

### Intention-to-treat analyses

#### Primary outcome

**Fig. 2:**
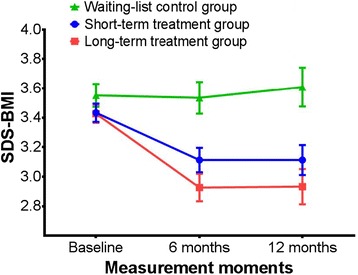
SDS-BMI for the short-stay and long-stay and waiting-list group during 12 months of follow-up according to the intention-to-treat principle. Error Bars indicate SE

Mean (SD) SDS-BMI was 3.4 (0.4) in both treatment groups at the start of the treatment. The course of SDS-BMI over time is graphically presented in Fig. 2. SDS-BMI decreased statistically significantly in the first six months in both groups. Participants were on average able to maintain this weight-loss during the second half year of treatment. SDS-BMI after 12 months of treatment was statistically significantly lower compared with baseline (mean difference (SD) 0.33 (0.48) in the short-stay and 0.52 (0.49) in the long-stay group). This decrease in SDS-BMI corresponds to an average (SD) weight-loss of 8.1 (14.3) kg in the short-stay and 12.6 (13.6) kg in the long-stay group.

Table 2 shows the crude and adjusted results of the GEE. Sex, ethnicity and socio economic status were included as covariates in the adjusted analyses. After six months of treatment, SDS-BMI of participants in the short-stay group was higher compared with the long-stay group (adjusted models β = 0.23, 95 % CI 0.09; 0.36). However, after one year of treatment, there was no statistically significant difference in SDS-BMI any more between the two treatment groups (adjusted models β = 0.15, 95 % CI −0.06; 0.35).

**Table 2:** Effects on outcomes after six and 12 months of follow-up for both treatment groups according to the intention-to-treat principle

	Short-stay	Long-stay	Crude	Adjusted
	Mean (SD)	Mean (SD)	Beta (95 % CI)	Beta (95 % CI)
Primary outcome measure				
SDS-BMI				
Baseline	3.4 (0.4)	3.4 (0.4)		
6-months	3.1 (0.5)	2.9 (0.6)	**0.22 (0.09; 0.35)**	**0.23 (0.09; 0.36)**
12-months	3.1 (0.6)	2.9 (0.7)	0.18 (−0.03; 0.40)	0.18 (−0.04; 0.40)
Overall effect^a^			0.10 (−0.11; 0.31)	0.15 (−0.06; 0.35)
Secondary outcomes				
SDS-waist circumference				
Baseline	3.2 (0.4)	3.1 (0.4)		
6-months	2.7 (0.5)	2.5 (0.5)	0.13 (−0.03; 0.30)	0.12 (−0.05; 0.30)
12-months	2.7 (0.7)	2.5 (0.7)	0.08 (−0.18; 0.34)	0.02 (−0.23; 0.27)
Overall effect^a^			0.14 (−0.07; 0.35)	0.15 (−0.07; 0.37)
Systolic blood pressure (mmHg)				
Baseline	122.7 (12.3)	121.1 (13.4)		
6-months	117.1 (13.9)	116.7 (13.4)	−0.63 (−4.73; 3.48)	0.17 (−3.98; 4.33)
12-months	118.4 (11.2)	120.0 (15.3)	−2.84 (−7.27; 1.60)	−2.25 (−6.95; 2.45)
Overall effect^a^			0.59 (−4.65; 5.82)	0.44 (−4.86; 5.75)
Diastolic blood pressure (mmHg)				
Baseline	75.7 (9.9)	78.0 (12.3)		
6-months	68.5 (7.9)	68.3 (11.2)	1.49 (−2.04; 5.02)	0.47 (−3.17; 4.11)
12-months	68.6 (8.1)	67.1 (12.0)	3.02 (−0.72; 6.76)	2.35 (−1.64; 6.34)
Overall effect^a^			−0.42 (−4.40, 3.56)	−1.21 (−5.21, 2.80)
Fasting insulin (μU/L)				
Baseline	13.8 (9.3)	14.6 (9,5)		
6-months	11.9 (9.3)	12.9 (10.8)	−0.98 (−4.50; 2.55)	−1.41 (−5.20; 2.39)
12-months	11.7 (9.1)	14.1 (10.1)	−1.27 (−4.25; 1.70)	−2.15 (−5.39; 1.09)
Overall effect^a^			−1.10 (−3.60; 1.40)	−2.30 (−5.75; 1.16)
2 h-insulin (μU/L)				
Baseline	70.7 (49.9)	60.8 (36.8)		
6-months	63.0 (56.9)	52.4 (37.7)	1.16 (−17.21; 19.52)	−0.80 (−19.82; 18.22)
12-months	67.9 (47.4)	65.3 (54.5)	−0.54 (−23.38; 22.48)	−2.89 (−26.49; 20.71)
Overall effect^a^			0.65 (−15.50; 16.80)	1.86 (−13.59; 17.31)
Fasting glucose (mmol/L)				
Baseline	4.7 (0.4)	4.7 (0.3)		
6-months	4.8 (0.4)	4.8 (0.3)	0.03 (−0.11; 0.16)	0.02 (−0.13; 0.16)
12-months	4.8 (0.4)	4.9 (0.3)	−0.06 (−0.19; 0.08)	−0.01 (−0.13; 0.12)
Overall effect^a^			−0.04 (−0.16; 0.08)	−0.02 (−0.14; 0.10)
2 h-glucose (mmol/L)				
Baseline	6.0 (1.2)	5.6 (1.1)		
6-months	5.4 (1.1)	5.1 (1.0)	−0.02 (−0.47; 0.42)	−0.02 (−0.51; 0.47)
12-months	5.8 (1.1)	5.3 (1.4)	0.32 (−0.24; 0.87)	0.29 (−0.32; 0.89)
Overall effect^a^			0.35 (−0.06; 0.77)	0.35 (−0.07; 0.76)
HDL-cholesterol (mmol/L)				
Baseline	1.1 (0.3)	1.0 (0.2)		
6-months	1.2 (0.3)	1.1 (0.2)	0.04 (−0.02; 0.11)	0.04 (−0.03; 0.11)
12-months	1.2 (0.3)	1.2 (0.3)	−0.07 (−0.17; 0.04)	−0.04 (−0.14; 0.07)
Overall effect^a^			0.07 (−0.03; 0.17)	0.09 (−0.02; 0.20)
Triglycerides (mmol/L)				
Baseline	1.0 (0.6)	1.0 (0.5)		
6-months	1.1 (1.0)	1.0 (0.5)	0.09 (−0.14, 0.31)	0.04 (−0.15, 0.22)
12-months	1.2 (0.9)	1.1 (0.7)	0.09 (−0.18, 0.35)	0.04 (−0.22, 0.29)
Overall effect^a^			0.09 (−0.18, 0.37)	0.06 (−0.17, 0.29)
HOMA-IR				
Baseline	2.9 (2.0)	3.1 (2.1)		
6-months	2.5 (2.0)	2.8 (2.4)	−0.17 (−0.96; 0.61)	−0.26 (−1.11; 0.59)
12-months	2.5 (2.0)	3.1 (2.4)	−0.33 (−1.02, 0.37)	−0.44 (−1.17, 0.30)
Overall effect^a^			−0.31 (−1.11; 0.49)	−0.52 (−1.28; 0.25)

Adjusted models corrected for baseline, sex, ethnicity, and economic status

*Abbreviations*: *CI* confidence interval, *SD* standard deviation, *SDS-BMI* standard deviation of body mass index, *SDS-waist circumference*standard deviation of waist circumference, *HDL* high-density lipoprotein, *HOMA-IR* homeostasis model assessment for insulin resistance

^a^Overall effect can be interpreted as the average difference over time between the two treatment groups

#### Secondary outcomes

Statistically significant improvements were seen after one year of treatment compared with baseline in both treatment groups in SDS-waist circumference, diastolic blood pressure and HDL-cholesterol. In the short-stay group, systolic blood pressure improved statistically significantly in comparison with baseline as well.

In the crude and adjusted GEE, no statistically significantly differences in secondary outcomes between the short-stay and long-stay groups were found at any point in time (Table 2).

### Analyses stratified by sex

#### Primary outcome

Sex was identified as an effect modifier. The course of SDS-BMI over time for boys and girls in the short-stay group followed the same course as described under the intention-to-treat analyses. However, in the long-stay group, SDS-BMI in boys decreased even further in the second six months of treatment, whereas girls showed an increase in SDS-BMI.

Results of the crude and adjusted GEE stratified by sex are reported in Tables 3 (boys) and 4 (girls). Only for girls, SDS-BMI of participants in the short-stay group was higher compared with the long-stay group after six months of treatment, (adjusted models β = 0.20, 95 % CI 0.07; 0.33). After one year of treatment, there was no statistically significant difference in SDS-BMI between the two treatment groups for either sex.

**Table 3:** Effects on outcomes after six and 12 months of follow-up for both treatment groups according to the intention-to-treat principle, boys only

	Short-stay	Long-stay	Crude	Adjusted
	Mean (SD)	Mean (SD)	Beta (95 % CI)	Beta (95 % CI)
Primary outcome measure				
SDS-BMI				
Baseline	3.7 (0.4)	3.7 (0.4)		
6-months	3.4 (0.6)	3.1 (0.7)	0.25 (−0.03; 0.52)	0.23 (−0.07; 0.53)
12-months	3.4 (0.7)	2.9 (0.8)	0.34 (−0.09; 0.77)	0.31 (−0.14; 0.76)
Overall effect^a^			0.25 (−0.15; 0.64)	0.26 (−0.15; 0.67)
Secondary outcomes				
SDS-waist circumference				
Baseline	3.5 (0.4)	3.3 (0.3)		
6-months	3.0 (0.7)	2.6 (0.6)	0.10 (−0.35; 0.55)	0.05 (−0.39; 0.48)
12-months	3.0 (1.0)	2.4 (0.9)	0.12 (−0.48; 0.72)	0.01 (−0.57; 0.60)
Overall effect^a^			0.40 (−0.03; 0.83)	0.40 (−0.06; 0.86)
Systolic blood pressure (mmHg)				
Baseline	124.3 (15.0)	125.1 (14.0)		
6-months	122.0 (18.7)	120.2 (11.9)	3.34 (−5.35; 12.03)	4.31 (−3.56; 12.19)
12-months	122.1 (12.3)	123.3 (14.4)	−1.46 (−9.12; 6.20)	0.45 (−6.74; 7.64)
Overall effect^a^			0.52 (−9.22; 10.25)	2.04 (−8.27; 12.36)
Diastolic blood pressure (mmHg)				
Baseline	77.7 (11.0)	80.9 (9.2)		
6-months	64.4 (8.0)	68.2 (10.0)	−1.61 (−8.46; 5.24)	−2.91 (−9.17; 3.35)
12-months	69.4 (8.3)	66.5 (11.7)	5.28 (−0.21; 10.76)	3.86 (−2.23; 9.94)
Overall effect^a^			−0.73 (−7.66, 4.06)	−1.80 (−7.66, 4.06)
Fasting insulin (μU/L)				
Baseline	15.3 (7.7)	16.2 (13.2)		
6-months	12.4 (12.6)	11.8 (11.0)	0.78 (−4.44; 6.00)	0.73 (−4.54; 6.00)
12-months	11.1 (5.8)	14.4 (13.8)	−0.82 (−4.84; 3.20)	−1.15 (−5.06; 2.76)
Overall effect^a^			−0.58 (−7.69; 6.53)	0.14 (−6.93; 7.22)
2 h-insulin (μU/L)				
Baseline	75.4 (50.2)	53.3 (34.6)		
6-months	64.5 (68.2)	38.6 (20.2)	13.21 (−21.43; 47.84)	14.30 (−18.69; 47.29)
12-months	65.1 (41.3)	62.7 (33.8)	2.81 (−14.94; 20.56)	3.47 (−16.01; 22.96)
Overall effect^a^			21.31 (−7.19; 49.81)	22.60 (−5.09; 50.30)
Fasting glucose (mmol/L)				
Baseline	4.6 (0.2)	4.8 (0.4)		
6-months	4.8 (0.3)	4.7 (0.3)	0.14 (−0.05; 0.34)	0.14 (−0.05; 0.34)
12-months	4.7 (0.3)	5.0 (0.2)	−0.16 (−0.36; 0.04)	−0.17 (−0.36; 0.02)
Overall effect^a^			−0.14 (−0.31; 0.03)	−0.10 (−0.25; 0.06)
2 h-glucose (mmol/L)				
Baseline	6.3 (1.1)	5.7 (0.9)		
6-months	5.7 (1.5)	4.9 (0.9)	0.43 (−0.47; 1.33)	0.48 (−0.39; 1.34)
12-months	5.6 (0.9)	6.0 (1.1)	−0.40 (−1.06; 0.27)	−0.43 (−1.14; 0.29)
Overall effect^a^			0.42 (−0.26; 1.10)	0.49 (−0.25; 1.23)
HDL-cholesterol (mmol/L)				
Baseline	1.1 (0.3)	1.0 (0.2)		
6-months	1.1 (0.4)	1.1 (0.2)	−0.08 (−0.18; 0.13)	−0.08 (−0.19; 0.03)
12-months	1.2 (0.5)	1.2 (0.4)	−0.08 (−0.27; 0.11)	−0.07 (−0.28; 0.15)
Overall effect^a^			0.05 (−0.17; 0.28)	0.08 (−0.13; 0.29)
Triglycerides (mmol/L)				
Baseline	1.0 (0.5)	1.2 (0.7)		
6-months	1.1 (0.7)	1.1 (0.7)	0.13 (−0.30, 0.56)	0.06 (−0.31, 0.44)
12-months	1.4 (0.9)	1.4 (0.9)	−0.06 (−0.51, 0.63)	−0.04 (−0.56, 0.48)
Overall effect^a^			−0.10 (−0.56, 0.36)	−0.10 (−0.52, 0.33)
HOMA-IR				
Baseline	3.1 (1.5)	3.5 (3.0)		
6-months	2.7 (2.7)	2.5 (2.4)	0.40 (−0.77; 1.58)	0.37 (−0.80; 1.54)
12-months	2.3 (1.2)	3.3 (3.3)	−0.17 (−1.05, 0.72)	−0.27 (−1.12, 0.59)
Overall effect^a^			−0.30 (−1.87; 1.28)	−0.12 (−1.68; 1.44)

Adjusted models corrected for baseline, ethnicity, and socio economic status

*Abbreviations*: *CI* confidence interval, *SD* standard deviation, *SDS-BMI* standard deviation of body mass index, *SDS-waist circumference* standard deviation of waist circumference, *HDL* high-density lipoprotein, *HOMA-IR* homeostasis model assessment for insulin resistance

^a^Overall effect can be interpreted as the average difference over time between the two treatment groups

**Table 4:** Effects on outcomes after 6 and 12 months of follow-up for both treatment groups according to the intention-to-treat principle, girls only

	Short-stay	Long-stay	Crude	Adjusted
	Mean (SD)	Mean (SD)	Beta (95 % CI)	Beta (95 % CI)
Primary outcome measure				
SDS-BMI				
Baseline	3.3 (0.3)	3.3 (0.3)		
6-months	3.0 (0.5)	2.8 (0.5)	**0.19 (0.06; 0.31)**	**0.20 (0.07; 0.33)**
12-months	3.0 (0.5)	3.0 (0.6)	0.06 (−0.16; 0.28)	0.07 (−0.14; 0.28)
Overall effect^a^			0.08 (−0.14; 0.30)	0.06 (−0.17; 0.30)
Secondary outcomes				
SDS-waist circumference				
Baseline	3.0 (0.3)	3.0 (0.3)		
6-months	2.6 (0.4)	2.5 (0.5)	0.10 (−0.05; 0.25)	0.10 (−0.06; 0.26)
12-months	2.6 (0.6)	2.6 (0.6)	−0.03 (−0.27; 0.22)	−0.07 (−0.28; 0.15)
Overall effect^a^			0.05 (−0.15; 0.26)	−0.00 (−0.22; 0.22)
Systolic blood pressure (mmHg)				
Baseline	122.0 (11.2)	118.7 (12.6)		
6-months	115.2 (11.5)	114.3 (14.1)	−2.25 (−6.63; 2.13)	−2.39 (−6.75; 1.96)
12-months	117.3 (10.9)	117.9 (15.9)	−3.71(−9.22; 1.81)	−4.17 (−10.08; 1.74)
Overall effect^a^			1.32 (−4.84; 7.49)	−0.27 (−6.22; 5.68)
Diastolic blood pressure (mmHg)				
Baseline	74.8(9.5)	76.3 (13.6)		
6-months	70.2 (7.5)	68.4 (12.2)	2.31 (−1.52; 6.13)	1.94 (−2.19; 6.07)
12-months	68.3 (8.3)	67.5 (12.5)	1.28 (−3.24; 5.80)	0.68 (−4.19; 5.55)
Overall effect^a^			−0.32 (−5.57, 4.92)	−0.56 (−5.86, 4.75)
Fasting insulin (μU/L)				
Baseline	13.2 (10.0)	13.6 (6.4)		
6-months	11.7 (7.8)	13.6 (10.9)	−2.18 (−6.87; 2.51)	−3.21 (−8.78; 2.37)
12-months	11.9 (10.2)	13.9 (7.2)	−1.68 (−5.66; 2.30)	−3.21 (−7.96; 1.55)
Overall effect^a^			−1.42 (−5.32; 2.48)	−3.68 (−6.88; 0.48)
2 h-insulin (μU/L)				
Baseline	68.7 (50.5)	65.5 (38.0)		
6-months	62.3 (52.9)	60.6 (43.5)	−6.39 (−28.67; 15.90)	−7.93 (−31.08; 15.22)
12-months	68.9 (50.4)	67.0 (65.4)	−2.70 (−37.22; 31.81)	−4.80 (−38.42; 28.82)
Overall effect^a^			0.16 (−32.31 21.64)	−10.37 (−27.09; 6.36)
Fasting glucose (mmol/L)				
Baseline	4.8 (0.4)	4.7 (0.2)		
6-months	4.8 (0.4)	4.8 (0.4)	−0.05 (−0.23; 0.12)	−0.09 (−0.28; 0.10)
12-months	4.8 (0.4)	4.8 (0.3)	0.01 (−0.15; 0.17)	0.06 (−0.09; 0.22)
Overall effect^a^			0.01 (−0.15; 0.16)	0.02 (−0.13; 0.17)
2 h-glucose (mmol/L)				
Baseline	5.9 (1.3)	5.6 (1.2)		
6-months	5.2 (1.0)	5.3 (1.1)	−0.26 (−0.77; 0.25)	−0.23 (−0.80; 0.34)
12-months	5.8 (1.2)	4.9 (1.4)	0.73 (−0.01; 1.48)	0.71 (−0.11; 1.54)
Overall effect^a^			0.34 (−0.18; 0.87)	0.21 (−0.24; 0.66)
HDL-cholesterol (mmol/L)				
Baseline	1.1 (0.2)	1.0 (0.2)		
6-months	1.2 (0.2)	1.1 (0.2)	**0.11 (0.03; 0.19)**	**0.11 (0.03; 0.19)**
12-months	1.2 (0.2)	1.2 (0.3)	−0.07 (−0.18; 0.05)	−0.03 (−0.14; 0.09)
Overall effect^a^			0.08 (−0.02; 0.18)	0.10 (−0.01; 0.21)
Triglycerides (mmol/L)				
Baseline	1.0 (0.6)	0.9 (0.3)		
6-months	1.1 (1.1)	0.9 (0.3)	−0.01 (−0.24, 0.21)	−0.05 (−0.25, 0.15)
12-months	1.1 (1.0)	0.9 (0.3)	0.08 (−0.16, 0.32)	0.03 (−0.19, 0.25)
Overall effect^a^			0.22 (−0.09, 0.54)	0.11 (−0.13, 0.36)
HOMA-IR				
Baseline	2.8 (2.3)	2.9 (1.4)		
6-months	2.5 (1.7)	2.9 (2.4)	−0.49 (−1.53; 0.55)	−0.73 (−1.96; 0.51)
12-months	2.5 (2.2)	3.0 (1.6)	−0.40 (−1.31, 0.51)	−0.63 (−1.72, 0.46)
Overall effect^a^			−0.29 (−1.13; 0.55)	−0.75 (−1.46; 0.04)

Adjusted models corrected for baseline, ethnicity, and socio economic status

*Abbreviations*: *CI* confidence interval, *SD* standard deviation, *SDS-BMI* standard deviation of body mass index, *SDS-waist circumference* standard deviation of waist circumference, *HDL* high-density lipoprotein, *HOMA-IR* homeostasis model assessment for insulin resistance

^a^Overall effect can be interpreted as the average difference over time between the two treatment groups

#### Secondary outcomes

Analyses stratified by sex showed statistically significant improvements in SDS-waist circumference, diastolic blood pressure and HDL-cholesterol after one year of treatment compared with baseline.

For girls there was a statistically significant difference in HDL-cholesterol between the short-stay and long-stay group after six months of follow-up (adjusted models β = 0.11, 95 % CI 0.03; 0.19). There were no statistically significant differences in secondary outcomes between the treatment groups after one year of treatment after stratification by sex as demonstrated by the crude and adjusted GEE (Tables 3 and 4).

### Additional analyses

#### Waiting-list group

During the waiting list period, mean (SD) SDS-BMI increased from 3.55 (0.31) to 3.61 (0.53) in the year prior to the start of treatment, but this was not statistically significant (Fig. 2).

The crude and adjusted GEE showed that the SDS-BMI of participants in the short-stay group and long-stay group was statistically significantly lower (0.32, 95 % CI −0.43; −0.21 and 0.49, 95 % CI −0.62; −0.36, respectively) after one year of treatment compared with the waiting-list group.

There was a statistically significant improvement in SDS-waist circumference, systolic- and diastolic blood pressure, fasting insulin, HDL-cholesterol, and HOMA-IR after one year of treatment in the two treatments groups in comparison with the waiting-list group (data not shown).

##### Per protocol and complete case analyses

In the additional analyses the course of SDS-BMI over time followed the same course in the per protocol analyses as in the intention-to-treat analyses.

After one year of treatment, there was no statistically significant difference in SDS-BMI between the two treatment groups (data not shown).

There were statistically significant improvements in both treatment groups in SDS-waist circumference, diastolic blood pressure and HDL-cholesterol after one year of treatment compared with baseline. In the short-stay group, systolic blood pressure improved statistically significantly in comparison with baseline as well. Additionally, in the per protocol analyses, in the short-stay group also fasting insulin, 2h-glucose and HOMA-IR improved statistically significantly.

In the complete case analyses, after one year of treatment there were no statistically significant differences between the treatment groups in any of the secondary outcomes; in the per protocol analyses there was a statistically significant difference between the treatment groups in HDL-cholesterol (data not shown).

## Discussion

In this randomized controlled trial, two intensive lifestyle treatments for severely obese children and adolescents with varying inpatient periods (two or six months) were compared. Both treatments showed statistically significant improvements in SDS-BMI and cardiometabolic risk factors after treatment compared with baseline. However, after one year of treatment, there were no statistically significant differences in SDS-BMI or cardiometabolic risk factors between the two treatment groups. In an additional analysis, it was shown that SDS-BMI and cardiometabolic risk factors of participants in both treatment groups improved statistically significantly after one year of treatment compared with participants in a waiting-list controlgroup.

The decreases in SDS-BMI that were observed in this study correspond to an average (SD) weight-loss of 8.1 (14.3) kg in the short-stay and 12.6 (13.6) kg in the long-stay group, which is generally considered a clinically relevant improvement [37]. The children in this study were referred to the specialized childhood obesity center because they did not respond to treatment in an ambulatory setting. This study shows that even in this children considerable weight loss can and improvement in cardiovascular risk be achieved.

Many of the participants’ parents were of Non-Western origin and had a lack of proficiency in the Dutch language which complicated treatment in some cases due to communication problems. Despite this, considerable improvements in SDS-BMI were achieved. Also, a relatively high percentage of the participants came from single-parent families and had a low SES background which can be considered a less favorable home environment to retain weight loss. However, our results showed that weight reductions achieved after the intensive inpatient treatment period in the first six months were on average maintained in the second half of the year when only monthly follow-up visits were provided [42–44].

Ambulatory obesity treatment often seems far less effective for severely obese children in comparison with children and adolescents with a lesser degree of obesity [26–28, 45]. Therefore, an inpatient treatment seems more appropriate for these children and adolescents [30]. An inpatient setting provides a more supportive environment than ambulatory obesity treatment where children often have to deal with a less supportive home environment every day. The large decrease in SDS-BMI we observed might therefore be explained by the extensive inpatient period in the treatments in our study. During the past decades in the Netherlands several studies have evaluated the effects of treatments for children and adolescents with (severe) obesity. Most of these studies, however, evaluated ambulatory treatment programs and included populations that consisted of a combination of obese and severely obese participants. A Dutch study by Hofsteenge et al. among severely obese children that were on average less obese than the participants followed in our study, showed that SDS-BMI after 18 months of treatment was statistically significantly lower in the intervention group than in the control group. However, this effect was only observed in obese adolescents from Western origin and not in those of Non-Western origin [46].

Another Dutch study by Vos et al. followed a group of severely obese children and adolescents receiving ambulatory obesity treatment for three months [47]. Directly after treatment, SDS-BMI had decreased and after one year of follow-up in which 2–3 refresher sessions were offered, SDS-BMI decreased even further [47]. The decrease in SDS BMI after one year was comparable to the decrease in SDS-BMI in our study after six months after which SDS-BMI stabilized. Only among boys in the long-stay group we also found a further decrease in SDS BMI in the second half year. A possible explanation for this difference in favor of severely obese boys, could be that puberty may contribute to a more beneficial weight development in boys [48].

The only other study by van der Baan et al. in the Netherlands that also evaluated treatment with an inpatient period of six months among severely obese children using the same inclusion criteria as our study, showed a decrease in SDS-BMI after six months that was comparable with our study. However, this study showed a slight increase in SDS-BMI in the second half year of follow-up [30]. This might be due to the fact that there were no return visits after the six-month inpatient period.

Research among severely obese children and adolescents is relatively rare. The few studies performed in the Netherlands and other countries show that among severely obese adolescents, improvements in SDS-BMI during obesity treatment can be maintained during follow-up [49]. However, sustained behavior change is a difficult and complex process. Therefore, it is important to also have sufficient support available for parents and children after the most intensive treatment period to maintain learned changes. Regular return visits or refresher sessions seem essential to prevent relapse.

### Strengths and limitations

This study has several strengths. First, it is unique with regard to the intensity of the treatments studied, since most lifestyle treatments for severely obese children and adolescents do not include an inpatient period. Moreover, although the duration of treatment was long with one year, this did not result in a high attrition rate; only 12 participants (15 %) dropped out of treatment. Compared to other studies evaluating the effects of obesity treatment in adolescents this dropout is rather small [46]. Because participants will go back to their home environment after treatment, it is very important that the family of the participants is involved in the treatment. Therefore, a second strength of this study is that not only the participants were involved in the treatment, but that the treatment was family-based with active parental participation. Finally, both treatment groups were compared to a waiting-list controlgroup. Therefore, this study not only gives insight into the effects of the intensive treatments compared to each other, but also as compared with a waiting-list condition during which the children received usual care.

There are several limitations as well. The treatments pose a high burden on the participating families. The frequent visits resulted in high time investments for parents and associated time costs. Moreover, parents/caregivers often needed to take time off from work to participate in treatment resulting in productivity losses. In addition, children were placed in an environment completely new to them and were away from their families, home, school and friends for an extended period of time, especially in the long-stay group. Another limitation is that, although a waiting-list group was included, data were available for only 16 participants as four participants in the waiting list condition dropped out of the study. Due to practical reasons, we were unable to recruit more participants into the waiting-list group prior to treatment.

### Implications

To ensure long-term maintenance of weight-loss after intensive treatment, continuous monitoring and periodic intensive return visits seem essential. Treatment in specialized childhood obesity centers is costly and poses a high burden to families and care-givers, so preferably long-term treatment is organized in the home environment making it more feasible and less expensive than long-term treatment in specialized childhood obesity centers. It is important that the organization and transfer of treatment from an inpatient setting to an ambulatory setting is prepared carefully, to ensure that participants are not lost to follow-up and receive the right kind of continuous treatment. Special attention to attrition from the continuous treatment in the ambulatory setting is needed.

## Conclusions

No statistically significant differences in SDS-BMI were found between the short-stay and long-stay inpatient segments during one year of treatment. However, both treatments resulted in statistically significant improvements in comparison with baseline and were statistically significantly more effective than a waiting list condition.

Based on these results, we recommend implementation of the short-stay treatment because of the lower burden for the participating families and the lower costs of treatment. However, whether this treatment should be implemented on a wider scale depends on whether the effects of the inpatient treatment in comparison with usual care are sustained over a longer period of time.

## References

[1] Jackson-Leach R, Lobstein T (2006). Estimated burden of paediatric obesity and co-morbidities in Europe. Part 1. The increase in the prevalence of child obesity in Europe is itself increasing. Int J Ped Obes.

[2] Ogden CL, Carroll MD, Kit BK, Flegal KM (2012). Prevalence of obesity in the United States, 2009–2010. NCHS data brief.

[3] Wang Y, Lobstein T (2006). Worldwide trends in childhood overweight and obesity. Int J Ped Obes.

[4] Schonbeck Y, Talma H, van Dommelen P, Bakker B, Buitendijk SE, Hirasing RA (2011). Increase in prevalence of overweight in Dutch children and adolescents: a comparison of nationwide growth studies in 1980, 1997 and 2009. PLoS One.

[5] Skelton JA, Cook SR, Auinger P, Klein JD, Barlow SE (2009). Prevalence and trends of severe obesity among US children and adolescents. Acad Pediatr.

[6] van Dommelen P, Schonbeck Y, Van Buuren S, Hirasing RA (2014). Trend in a life threatening condition: morbid obesity in Dutch, Turkish and Maroccan children in the Netherlands. PLoS One.

[7] Franks PW, Hanson RL, Knowler WC, Sievers ML, Bennett PH, Looker HC (2010). Childhood obesity, other cardiovascular risk factors, and premature death. New Engl J Med.

[8] Reilly JJ, Methven E, McDowell ZC, Hacking B, Alexander D, Stewart L (2003). Health consequences of obesity. Arch Dis Child.

[9] Makkes S, Renders CM, Bosmans JE, van der Baan-Slootweg OH, Seidell JC (2013). Cardiometabolic risk factors and quality of life in severely obese children and adolescents in The Netherlands. BMC Pediatr.

[10] van Emmerik NM, Renders CM, van de Veer M, van Buuren S, van der Baan-Slootweg OH, Kist-van Holthe JE (2012). High cardiovascular risk in severely obese young children and adolescents. Arch Dis Child.

[11] Buttitta M, Iliescu C, Rousseau A, Guerrien A (2014). Quality of life in overweight and obese children and adolescents: a literature review. Qual Life Res.

[12] Falkner NH, Neumark-Sztainer D, Story M, Jeffery RW, Beuhring T, Resnick MD (2001). Social, educational, and psychological correlates of weight status in adolescents. Obes Res.

[13] Puder JJ, Munsch S (2010). Psychological correlates of childhood obesity. Int J Obes.

[14] van Wijnen LG, Boluijt PR, Hoeven-Mulder HB, Bemelmans WJ, Wendel-Vos GC (2010). Weight status, psychological health, suicidal thoughts, and suicide attempts in Dutch adolescents: results from the 2003 E-MOVO project. Obesity.

[15] Schwimmer JB, Burwinkle TM, Varni JW (2003). Health-related quality of life of severely obese children and adolescents. JAMA.

[16] Baker JL, Olsen LW, Sorensen TIA (2007). Childhood body-mass index and the risk of coronary heart disease in adulthood. New Engl J Med.

[17] Singh AS, Mulder C, Twisk JW, van Mechelen W, Chinapaw MJ (2008). Tracking of childhood overweight into adulthood: a systematic review of the literature. Obes Rev.

[18] Ho M, Garnett SP, Baur L, Burrows T, Stewart L, Neve M (2012). Effectiveness of lifestyle interventions in child obesity: systematic review with meta-analysis. Pediatrics.

[19] Ho M, Garnett SP, Baur LA, Burrows T, Stewart L, Neve M (2013). Impact of dietary and exercise interventions on weight change and metabolic outcomes in obese children and adolescents: a systematic review and meta-analysis of randomized trials. JAMA Pediatr.

[20] Logue J, Thompson L, Romanes F, Wilson DC, Thompson J, Sattar N (2010). Management of obesity: summary of SIGN guideline. BMJ.

[21] Oude Luttikhuis H, Baur L, Jansen H, Shrewsbury VA, O'Malley C, Stolk RP (2009). Interventions for treating obesity in children. Cochrane Database Syst Rev.

[22] Seidell JC, Halberstadt J, Noordam H, Niemer S (2012). An integrated health care standard for the management and prevention of obesity in The Netherlands. Fam Pract.

[23] Bocca G, Corpeleijn E, Stolk RP, Sauer PJ (2012). Results of a multidisciplinary treatment program in 3-year-old to 5-year-old overweight or obese children: a randomized controlled clinical trial. Arch Pediatr Adolsc Med.

[24] Karner-Rezek K, Knechtle B, Fenzl M, Schlegel C, Konrad M, Rosemann T (2013). The effects of an 8-week multicomponent inpatient treatment program on body composition and anaerobic fitness in overweight and obese children and adolescents. Int J Gen Med.

[25] Davis CL, Pollock NK, Waller JL, Allison JD, Dennis BA, Bassali R (2012). Exercise dose and diabetes risk in overweight and obese children: a randomized controlled trial. JAMA.

[26] Yanovski JA, Yanovski SZ (2003). Treatment of pediatric and adolescent obesity. JAMA.

[27] Barlow SE, Dietz WH (2002). Management of child and adolescent obesity: summary and recommendations based on reports from pediatricians, pediatric nurse practitioners, and registered dietitians. Pediatrics.

[28] Krebs NF, Himes JH, Jacobson D, Nicklas TA, Guilday P, Styne D (2007). Assessment of child and adolescent overweight and obesity. Pediatrics.

[29] Braet C, Tanghe A, Bode PD, Franckx H, Winckel MV (2003). Inpatient treatment of obese children: a multicomponent programme without stringent calorie restriction. Eur J Pediatr.

[30] Van der Baan-Slootweg OH, Benninga MA, Beelen A, Van der Palen J, Tamminga-Smeulders CLJ, Tijssen JGP (2014). Inpatient treatment of children and adolescents with severe obesity in the Netherlands; a randomized controlled trial. JAMA Pediatr.

[31] Makkes S, Halberstadt J, Renders CM, Bosmans JE, van der Baan-Slootweg OH, Seidell JC (2011). Cost-effectiveness of intensive inpatient treatments for severely obese children and adolescents in the Netherlands; a randomized controlled trial (HELIOS). BMC Public Pealth.

[32] Pocock S (1983). Clinical Trials: A Practical Approach.

[33] Random number generator: http://www.pangloss.com/seidel/rnumber.cgi.

[34] Cole TJ, Bellizzi MC, Flegal KM, Dietz WH (2000). Establishing a standard definition for child overweight and obesity worldwide: international survey. BMJ.

[35] Growth Analyser. Stichting Kind en Groei; 2010. http://kindengroei.nl/growth-analyser/

[36] Matthews DR, Hosker JP, Rudenski AS, Naylor BA, Treacher DF, Turner RC (1985). Homeostasis model assessment: insulin resistance and beta-cell function from fasting plasma glucose and insulin concentrations in man. Diabetologia.

[37] Ford AL, Hunt LP, Cooper A, Shield JP (2010). What reduction in BMI SDS is required in obese adolescents to improve body composition and cardiometabolic health?. Arch Dis Child.

[38] IBM Corp. IBM SPSS Statistics for Windows, Version 21.0. Armonk, NY: IBM Corp; 2012.

[39] Twisk JWR (2013). Applied Longitudinal Data Analysis for Epidemiology. A practical guide. second ed.

[40] Fitzmaurice GM, Laird NM, Ware JH (2004). Applied longitudinal data analysis.

[41] Sonis J (1998). A closer look at confounding. Fam Med.

[42] Kalarchian MA, Levine MD, Arslanian SA, Ewing LJ, Houck PR, Cheng Y (2009). Family-based treatment of severe pediatric obesity: randomized, controlled trial. Pediatrics.

[43] Rossen LM (2014). Neighbourhood economic deprivation explains racial/ethnic disparities in overweight and obesity among children and adolescents in the U.S.A. J Epidemiol Community Health.

[44] de Jong E, Visscher TL, HiraSing RA, Seidell JC, Renders CM (2015). Home environmental determinants of children's fruit and vegetable consumption across different SES backgrounds. Pediatr Obes.

[45] Eliakim A, Kaven G, Berger I, Friedland O, Wolach B, Nemet D (2002). The effect of a combined intervention on body mass index and fitness in obese children and adolescents - a clinical experience. Eur J Pediatr.

[46] Hofsteenge GH, Chinapaw MJ, de Waal HA D-v, Weijs PJ (2014). Long-term effect of the Go4it group treatment for obese adolescents: A randomised controlled trial. Clin Nutr.

[47] Vos RC, Wit JM, Pijl H, Houdijk EC (2011). Long-term effect of lifestyle intervention on adiposity, metabolic parameters, inflammation and physical fitness in obese children: a randomized controlled trial. Nutr Diabetes.

[48] Danielsson P, Kowalski J, Ekblom O, Marcus C (2012). Response of severely obese children and adolescents to behavioral treatment. Arch Pediatr Adolesc Med.

[49] Whitlock E, O'Connor E, Williams S, Beil T, Lutz K. Effectiveness of weight management programs in children and adolescents. Evidence report/technology assessment. Report No.: 08-E014. Rockville, MD; 2008. PMC4781137 19408967

